# Implementing care for women with gestational diabetes after delivery—the challenges ahead

**DOI:** 10.3389/fgwh.2024.1391213

**Published:** 2024-08-16

**Authors:** Pei Chia Eng, Ada Ee Der Teo, Tong Wei Yew, Chin Meng Khoo

**Affiliations:** ^1^ Department of Endocrinology, National University Health Systems, Singapore, Singapore; ^2^ Department of Medicine, Yong Loo Lin School of Medicine, National University of Singapore, Singapore, Singapore; ^3^ Department of Digestion, Metabolism and Reproduction, Imperial College London, London, United Kingdom

**Keywords:** gestational diabetes, postpartum, cardiovascular disease, impaired glucose tolerance, oral glucose tolerance test (OGTT)

## Abstract

Gestational diabetes (GDM), defined as glucose intolerance during pregnancy, affects one in six pregnancies globally and significantly increases a woman’s lifetime risk of type 2 diabetes mellitus (T2DM). Being a relatively young group, women with GDM are also at higher risk of developing diabetes related complications (e.g., cardiovascular disease, non-alcoholic fatty liver disease) later in life. Children of women with GDM are also likely to develop GDM and this perpetuates a cycle of diabetes, escalating our current pandemic of metabolic disease. The global prevalence of GDM has now risen by more than 30% over the last two decades, making it an emerging public health concern. Antepartum management of maternal glucose is unable to fully mitigate the associated lifetime cardiometabolic risk. Thus, efforts may need to focus on improving care for women with GDM during the postpartum period where prevention or therapeutic strategies could be implemented to attenuate progression of GDM to DM and its associated vascular complications. However, strategies to provide care for women in the postpartum period often showed disappointing results. This has led to a missed opportunity to halt the progression of impaired glucose tolerance/impaired fasting glucose to DM in women with GDM. In this review, we examined the challenges in the management of women with GDM after delivery and considered how each of these challenges are defined and could present as a gap in translating evidence to clinical care. We highlighted challenges related to postpartum surveillance, postpartum glucose testing strategies, postpartum risk factor modification, and problems encountered in engagement of patients/providers to implement interventions strategies in women with GDM after delivery. We reasoned that a multisystem approach is needed to address these challenges and to retard progression to DM and cardiovascular disease (CVD) in women with GDM pregnancies. This is very much needed to pave way for an improved, precise, culturally sensitive and wholistic care for women with GDM.

## 1 Introduction

**Figure 1:**
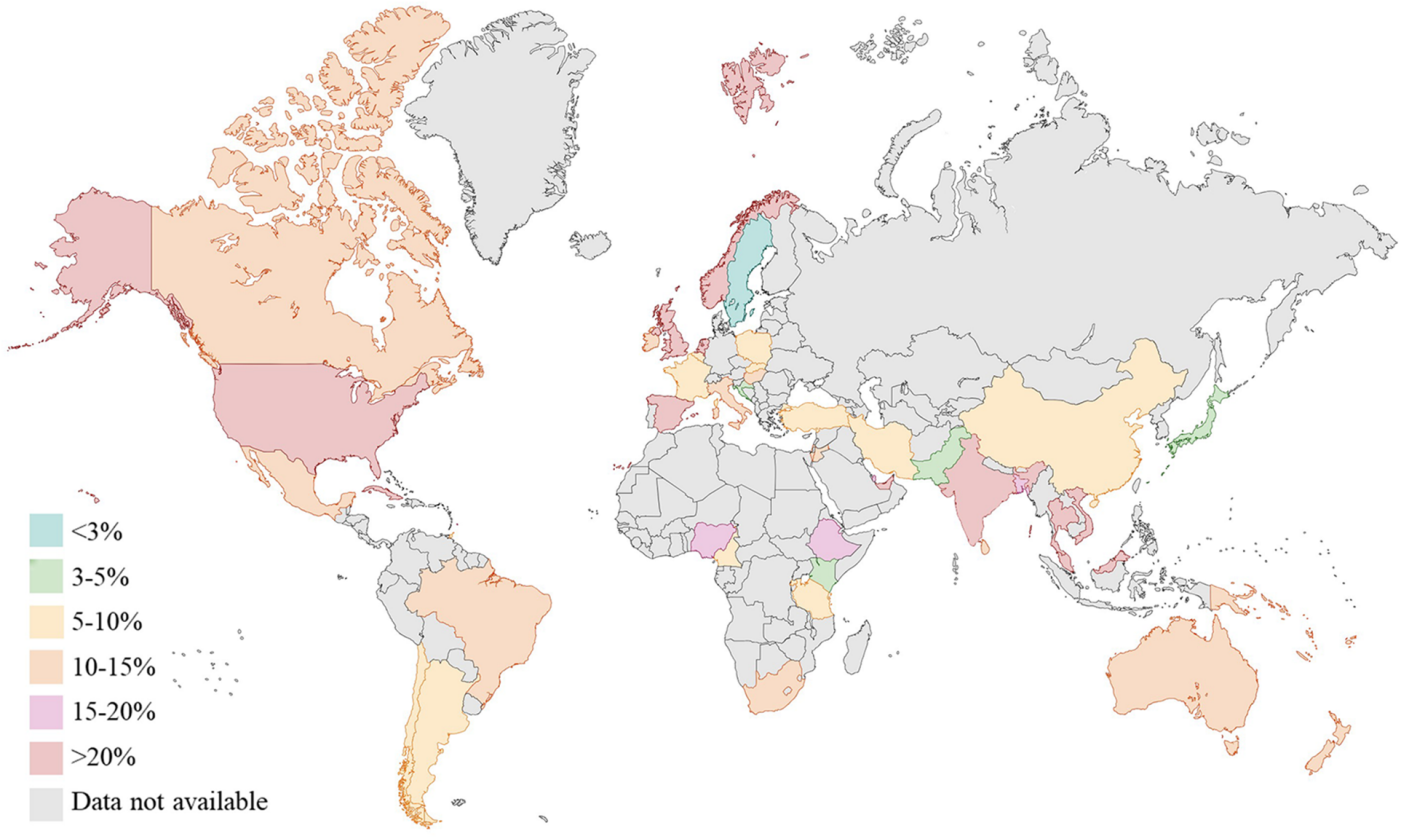
Prevalence (%) of gestational diabetes (GDM) worldwide (data from international diabetes federation atlas 2021). Created with Biorender.

Gestational diabetes (GDM), defined as glucose intolerance during pregnancy, has risen in prevalence by more than 30% across all population groups over the last two decades, giving rise to an emerging public health burden (1). Globally, GDM is known to affect one in six pregnancies, with higher prevalence in Middle East and North Africa (30.2%) and in South-east Asia (23.7%) (1) (Figure 1).

Compared to women without GDM, women with GDM have a ten-fold increased risk of developing type 2 diabetes (T2DM) after the index pregnancy (2). In women with GDM, the linear risk of progression to diabetes is 9.6% per year after delivery, with the risk being higher in the first 5 years after delivery (2, 3). Ethnicity modifies diabetes risk in different ethnic groups. Women of South Asians and Black ethnicity are associated with an increased absolute risk of T2DM compared to White (4). However, the relative incremental risk of progression from GDM to T2DM could actually be higher in White ethnic groups compared to women of Chinese and South Asian ethnicity (White: adjusted HR13.6; 95% CI 13.2,14.0), Chinese: adjusted HR9.2; 95% CI 8.1, 10.3; South Asian women: adjusted HR9.6; 95% CI 8.8, 10.5) (5). Additionally, women with GDM, despite being a relatively young cohort, have a two-fold increased risk of cardiovascular disease (CVD) (6) and non-alcoholic fatty liver disease (NAFLD) (7) after delivery. Children from women with GDM are more likely to be macrosomic at birth and have a greater propensity to develop obesity and T2DM later in life (8). Female offsprings are also likely to experience GDM in their own pregnancies resulting in a vicious intergenerational cycle of GDM (9).

**Figure 2:**
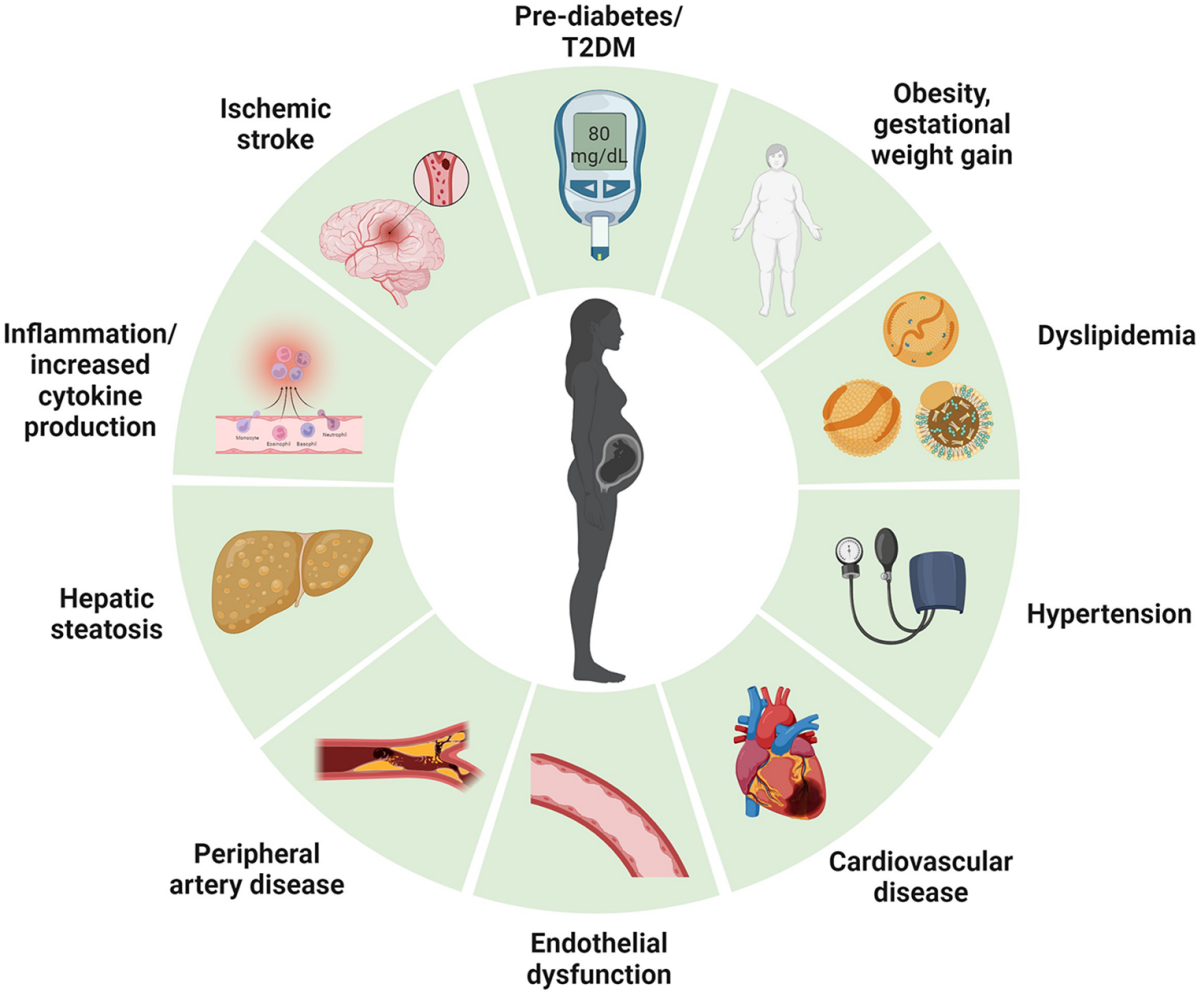
Long term cardiometabolic consequences of women with gestational diabetes mellitus. Created with Biorender.

Given that T2DM, CVD and NAFLD are significant sequels to GDM, close monitoring of postpartum GDM is essential to prevent the development of T2DM. This is because detection of dysglycaemia early in the trajectory of cardiometabolic disease could enable implementation of risk-modifying intervention that reduce the growing prevalence of diabetes (Figure 2) but also mitigate associated cardiometabolic complications. However, an optimal cost-effective program to identify, monitor and manage women with GDM with elevated cardiometabolic risk post-delivery is currently lacking. In this review, we aim to summarize the key challenges in managing the metabolic sequalae in women with GDM during the postpartum period.

## 2 Current challenges in postpartum management of women with GDM

### 2.1 Challenges in postpartum testing

#### 2.1.1 Is OGTT sufficient in stratifying glycaemic status postpartum?

The World Health Organisation (WHO) recommends a 75-grams oral glucose tolerance test (OGTT) as the screening test to reclassify glycaemic status in women with GDM after delivery (10). The OGTT involves a fasting glucose and a 2 h post-glucose load measurement and uses non-pregnancy criteria to identify women with impaired fasting glucose (IFG), impaired glucose tolerance (IGT), diabetes mellitus (DM), or normoglycaemia in the first 6 months after delivery (10). The IGT represents an intermediate state between normal and overt diabetes and individuals with IGT typically convert to T2DM at a rate of ∼5%–10% per year (11, 12). However, the risk of dysglycaemia could extend into women with normal glucose tolerance (NGT); 17.1% of women with GDM with NGT at 3 months postpartum developed prediabetes/diabetes within a year after delivery (13). Women with NGT who progressed to prediabetes/diabetes have higher fasting, 1 h and 2 h glucose level and tend to have a delayed peak blood glucose level at 60 min (16.1% of the progressors peak at 60 min on an OGTT compared to 6.5% of the progressors who peak at 30 min) (13). Conceivably, the defects in insulin secretion are likely to be a continuous process that begins long before the onset of overt diabetes. A ∼40%–50% loss in β-cell function is expected in women who had NGT with a 2 h OGTT of 6.6 mmol/L to 7.8 mmol/L (120–140 mg/dl) (14). Ravi Retnakaran et al. observed that women with mild glucose intolerance during pregnancy that do not meet criteria for diagnosis of GDM had β-cell dysfunction at 3–12 months postpartum (13, 15, 16), suggesting a progressive loss of β-cell function beyond pregnancy. Loss of β-cell function is likely to be independent of changes in adiposity or insulin sensitivity (16), highlighting a key pathophysiologic process that drives dysglycaemia (13, 17, 18) in women with GDM after delivery.

Most guidelines have recommended repeating OGTT in 1-year after delivery to re-stratify diabetes risk (19–21). Longitudinal studies consistently reported increased CVD and T2DM risk in women with NGT (6, 13) after delivery, thus a single 2 h OGTT measurement at 6–12 weeks postpartum may not have the sensitivity to identify women who are at high-risk for metabolic disease (22). Furthermore, OGTT is cumbersome, requires overnight fasting and additional staffing.

Abnormal glucose challenge test following an antepartum OGTT has been shown to predict pre-diabetes at 3 months postpartum with an AuROC of 0.754 in women with GDM compared to women with NGT during an antepartum OGTT (15). The glucose excursion during antepartum OGTT is a far more predictive metabolic marker compared to other metabolic measures such as the insulinogenic index or the homeostatic model assessment of insulin resistance (HOMA-IR) (15). Indeed, the number of abnormal OGTT values on a three-point OGTT test during pregnancy predicts the risk of developing T2DM at 5 years after the index pregnancy in a dose-response manner (23). A high fasting glucose during OGTT in pregnancy is strongly associated with development of T2DM in women with GDM compared to a high 2 h post-glucose load level (23). If glucose excursion values during pregnancy could provide insight into the future maternal risk of prediabetes (15), it would be reasonable to utilize it as a means to identify women at high-risk of glycemic and cardiometabolic deterioration in the postpartum period. This might be far more feasible especially when women rarely return for a postpartum OGTT test (24, 25) (described in sections below).

#### 2.1.2 Using 1-hour-post glucose level to predict diabetes and complications?

The 1 h plasma glucose level ≥8.6 nmol/L (155 mg/dl) during an OGTT may identify individuals with NGT at high risk of progressing to T2DM and CVD (26–28). A cohort study of 1945 non-diabetic men and women followed over 24 years showed that individuals with a 1 h prandial glucose of ≥8.6 mmol/L and a 2 h post-glucose level of <7.8 mmol/L had a 4.35-odds (95% CI 2.50−7.73) and a 1.87-odds (95% CI 1.09−3.26) of developing diabetes and prediabetes respectively (29). Elevated 1 h post glucose level of 8.6 mmol/L was also associated with an adverse cardiovascular risk profile characterised by higher blood pressure, elevated low-density lipoprotein, triglycerides and increased inflammatory markers and carotid intima thickness (30–32). In addition to macrovascular complications, 1 h plasma glucose of ≥8.6 mmol/L also predicted progression to microvascular complications, such as diabetic retinopathy and peripheral vascular complications, in individuals with NGT and IGT during 39 years follow-up (33). Compared to the 2 h post-glucose level, the 1 h post-glucose level of ≥8.6 mmol/L offered greater sensitivity in identifying a high-risk NGT group at an earlier time point before β-cell decline (22, 29, 33) in multiethnic groups (34–37) and predicted future diabetes better than fasting plasma glucose (FPG), 2 h plasma glucose, and HbA1c (AuROC of for 1 h plasma glucose of 0.84; AuROC for FPG 0.75; AuROC of 2 h plasma glucose is 0.79 and AuROC of HbA1c is 0.73) (27, 28, 38, 39).

The utility of 1 h post glucose value was endorsed by International Diabetes Federation (IDF) (40). In a recent position statement, individuals with 1 h post-glucose value of ≥8.6 mol/L were categorized as intermediate hyperglycaemia and should be commenced on lifestyle prevention program (40). People with 1 h post glucose level of ≥11.6 mmol/L were classified as T2DM and should have a repeat OGTT to confirm diagnosis (40). Overall, the accrued data suggested better stratification of risk of future T2DM, diabetes-related complications, and NAFLD with the 1 h post-glucose level of 8.6 nmol/L (40, 41). This would be of great relevance to women with GDM who are likely to have an underlying mild β-cell defect, which may not become apparent until years after pregnancy (20). The shortened OGTT procedure (from 2 h to 1 h) is also more cost-effective and clinically appealing to women with GDM who found the 2 h OGTT procedure to be time-consuming (40).

#### 2.1.3 Accuracy of other measures to assess glycaemic status in early postpartum period

Fasting plasma glucose (FPG) and HbA1c have been suggested as alternative screening tests to determine if a woman’s glucose status had returned to normal after delivery. FPG was correlated to HbA1c (*r* = 0.39) and the 2 h post-glucose value (*r* = 0.34) (42) but using FPG alone (at ≥6.1 mmol/L) resulted in missed diagnosis of impaired glucose tolerance (IGT) in 54% of women with GDM after delivery (43). In another study, 38.3% of women classified as glucose intolerance using OGTT test were reclassified as normal with a FPG (44). A postpartum FPG alone, whilst useful, may not be sensitive enough to ascertain glucose tolerance in high-risk multi-ethnic population (43), and is likely to lead to missed cases of diabetes and IGT.

Unlike FPG, HbA1c is relatively easy to perform but it could be affected by age, race, haematological factors or iron deficiency (45–48). HbA1c is not reliable in the first 1 year postpartum, due to blood loss during labour and persistence of high red cell turnover state (49). A HbA1c cut-off of 6.5% would misclassify 75% of the women with GDM who were previously categorized as abnormal glucose regulation by an OGTT test in the postpartum period (44). HbA1c is also weakly correlated with glycaemic parameters such as insulin sensitivity (*r* = −0.25, *p* = 0.010) or glucose disposition index (*r* = −0.26, *p* = 0.007) in women with GDM during early post-partum (3–6 months) (50). Using a lower HbA1c cutoff of ≥6% (42 mmol/mol) would increase the number of false negative that does not sufficiently identify IFG or IGT in postpartum GDM women (Specificity: 83.9%, 95% CI 73.2–92.9; Sensitivity: 23.8%, 95% CI 9.5–42.9) (50). Further lowering of the HbA1c cut-off to 5.7% would reduce its specificity (50). Notably, HbA1c 5.7−6.4% was a less precise predictor of glucose abnormalities in at risk individuals or in women with GDM in early postpartum period (42, 50) but could inform progression of glucose intolerance if assessed longitudinally and periodically during postpartum period (50). FPG could be used in combination with HbA1c in the prediction of diabetes during the postpartum period (51). A study from India showed that a FPG of ≥6.1 mmol/L or HBA1c ≥ 6.0% avoided OGTT in 80.9% of the women, without missing any cases of diabetes compared to missing 2.4% cases of diabetes when either FPG ≥5.6 mmol/L or HbA1c ≥ 5.7% were used alone (51).

#### 2.1.4 Lack of consensus in the guidelines on postpartum follow-up

Guidelines differ in terms of timing and the type of screening test for postpartum glycaemic status in women with GDM (Table 1).

**Table 1:** Postpartum oral glucose tolerance test (OGTT) guidelines for women with a history of GDM.

Organisation	ADIPS	Endocrine society	ADA	CDA	ACOG	UK NICE
Screening timeline	6–12 weeks postpartum	6–12 weeks postpartum	4–12 weeks postpartum, if normal, repeat OGTT every 1–3 years	6 weeks–6 months postpartum	4–12 weeks postpartum, if normal, repeat every 1 to 3 years	6–13 weeks postpartum, if normal, repeat annually
Screening test	75 g 2 h OGTT	75 g 2 h OGTT	75 g 2 h OGTT (HbA1c not recommended at 4–12 weeks postpartum	75 g 2 h OGTT	FPG or 75 g 2 h OGTT	FPG, HbA1c (75 g 2 h OGTT not recommended)
			Ongoing evaluation with HbA1c, FPG, 75 g 2 h OGTT			

ADIPS, Australasian diabetes in pregnancy society; ADA, American diabetes association; CDA, Canadian diabetes association; ACOG, American college of obstetricians and gynaecologists; UK NICE, United Kingdom national institute for health and care excellence; FPG, fasting plasma glucose.

The Australasian Diabetes in Pregnancy Society (52) and Endocrine Society (53) recommend screening for type 2 diabetes in women with previous GDM at least 6–12 weeks postpartum with a 75-g oral glucose tolerance test (OGTT), using non pregnancy criteria. The American Diabetes Association recommends screening for T2DM with an OGTT at an earlier time frame (4–12 weeks after delivery) to enable discussion of result at the 6-week postpartum obstetrical assessment (49, 54), whereas the Canadian Diabetes Association (CDA) suggests the same test over a longer period of assessment (from 6 weeks to 6 months) (19). The American Congress of Obstetrician and Gynaecologist indicates screening with either the OGTT or testing with fasting plasma glucose (FPG) at 6–12 weeks postpartum (21). On the other hand, the National Institute of Health and care Excellence (NICE) excludes a routine OGTT and suggests testing with a FPG or HbA1c at 6–13 weeks postpartum if FPG is not done earlier at discharge (55). The substantial variation in clinical recommendations throughout the world has made it challenging to understand the trajectory of cardiovascular and metabolic risk of women with GDM after pregnancy.

### 2.2 Challenges in adherence to postpartum testing

#### 2.2.1 Adherence to post-partum OGTT (patient and provider’s perspective)

Despite the clinical relevance of OGTT in classifying postpartum dysglycaemia, uptake of postpartum OGTT has been universally low globally, ranging from 31%–49% in most studies (56–58). This is much lower compared to postnatal cervical screening (94%) and antenatal GDM screening (98%) (59).

Both patients and providers have highlighted several barriers to postpartum OGTT. Bennett et al. conducted a semi-structured interviews in women with GDM and identified several themes of barriers to postpartum OGTT testing, which include: (1) emotional stress of prioritizing newborn’s needs before a woman’s postpartum care needs, the challenging adjustment to the new role as a mother and fear of receiving a diagnosis of diabetes, (2) lack of communication from providers resulting in underappreciation of the condition and a perceived sense of lack of continuity of care due to change of healthcare providers (60–62). Interestingly, the barriers reported were largely congruent across different ethnic groups (61, 63). Hewage SS et al. conducted an exploratory study in Singapore and found that despite universal GDM education, 37% of the women with GDM did not feel that postpartum OGTT was very important (61). The time-consuming nature of the OGTT test, the unpleasant taste of the glucose drink, inadequate education on postnatal care and lack of communication from relevant healthcare providers were highlighted as common barriers to postpartum OGTT amongst women with GDM in Singapore (61). Similarly, women with GDM of Hispanic, African American and White ethnic group would not adopt behaviour change before a subsequent pregnancy because they did not view prevention of GDM in future pregnancy as *a priori*ty (63). Although GDM was often seen as an important “wake-up” call for action, healthy behaviour change after pregnancy was typically not sustained (61, 63) could also influence motivation for sustained behaviour change. In Singapore, cultural practices such as confinement diet [diet consisting of red date tea (high sugar content) and herbal soups] for 14–40 days after delivery resulted in women consuming more refined carbohydrates and indulging in cravings after confinement period (64). Thus, addressing the perceived beliefs regarding continuation of health behaviours after childbirth is crucial in a successful postpartum program (65) (Table 2).

**Table 2:** Challenges in the management of women with gestational diabetes after delivery.

Patient factors	Provider factors	System factors	Process factors
Risk perception of progression of GDM to type 2 diabetes is low	Lack of time to communicate to patients on risk.	Lack of a channel for cross provider communication	Lack of a seamless workflow pattern between different specialties
Misunderstanding of advice given by different healthcare professionals	Unclear responsibility of follow-up on postpartum screening and care.	Lack of robust way of data tracking	Lack of cost-effective data on a diabetes prevention program
Emotional demands due to new role of being a mother	Risk perception of progression of GDM to T2DM is low amongst some providers	Inability to appreciate a shift in model of care from focusing on women in reproductive years to care for women and metabolic health over time.	Lack of infrastructure to support care:
			e.g., patient registry for women with GDM
Logistics—lack of time to attend clinic for an OGTT test; childcare issues	Different international diagnostic criteria of GDM and different screening and testing approaches postpartum		
Unpleasant taste of OGTT and the longer procedure involved compared to a single blood test.	Insufficient scientific data on the most appropriate treatment strategy		
	Incomplete evaluation of other metabolic parameters such as lipid profile in this group of women.		

From the healthcare providers’ perspective, challenges in postpartum OGTT include lack of familiarity of screening protocols, attitudinal barriers such as having patients underestimating the severity of T2DM and perceiving the postpartum OGTT as unnecessary or costly (66). Even more worryingly, a study reported 49% of the incomplete OGTT was attributed to providers not requesting the test (67) (Table 2).

#### 2.2.2 Uncertainty between primary and secondary care for postpartum screening

A challenge in the management of postpartum GDM is the lack of clear directions as to who should bear the responsibility of postpartum care for women. In some countries, the primary care providers (68) are expected to follow up women with GDM with a postpartum OGTT, whereas in other countries, internists are involved in the postpartum care for women with GDM (69). In practice, the type of tests to be used in assessment of glycaemic status after childbirth, frequency and duration of follow up deviated from national guidelines (70). Most specialists (73%) recommended long-term postpartum follow up but only 39% of primary care providers recalled women with GDM for diabetes screening (70).

Fragmentation of health services is a major barrier to postpartum screening (68, 70, 71). Hewage SS et al. pointed out that women were more likely to comply to T2DM preventive measures if recommended by healthcare providers (61). However, including a postpartum specialist clinician visit did not always result in higher rates of postpartum OGTT completion (56), particularly if women with GDM were not motivated to return for postpartum screening. Of the 81.1% of women who had postpartum clinician visit, 52% did not have a postpartum OGTT despite being arranged for them prior to presentation to a postpartum clinic (56). This suggests that the way the message was framed and delivered could influence a women’s decision to adhere to postpartum healthy behaviours (61).

In some countries, establishing a registry of women with previous GDM was expected to improve uptake of postpartum OGTT (72) but real-life data on the effectiveness of the GDM registry is not yet known. In Australia, the gestational diabetes registry had facilitated the process of sending automatic reminders for women with GDM to attend pre-booked postpartum OGTT screening, leading to a 9% increase in postpartum OGTT testing (73). Using a registry to recall women with GDM into primary care for postpartum screening was also shown to be effective, suggesting a potential utility of incorporating GDM register into family practice (74) (Table 2).

#### 2.2.3 Interventions to improve OGTT uptake may not be translatable in clinical practice

Various measures have been undertaken to overcome the barriers to postpartum OGTT testing. These include patient reminders in the form of postal (75), email or phone messages (76), verbal and written antepartum counselling, flexible appointment times, advanced order sets for glucose monitoring at 35 weeks pregnancy visit, educational modules to increase awareness amongst women regarding metabolic risk (77). Whilst all these measures show reasonable improvement in the uptake of postpartum OGTT in clinical studies, changes in postpartum OGTT screening rates in clinical practice outside the context of clinical studies were minimal (24). This suggests a gap in the translation of research to healthcare practice. Involvement of other healthcare professionals, such as nurses or case managers, seems to improve postpartum OGTT adherence (25, 78). As seen in the Women in India with Gestational Diabetes Strategies (WINGS) project in India, it is possible to obtain a 95.8% (203/212) postpartum follow-up rate through sustained efforts by trained healthcare professionals to contact women (79). Aside from periodic reminders, strategies such as offering postpartum screening to women with GDM during child immunization visits and integrating GDM screening with national public health programs have also been suggested (80). An electronic self-administered capillary OGTT device was reported to have good user-applicability by untrained individuals in community and could be tested as a screening tool in women with GDM in future (81).

Mobile applications such as smartphones and mobile apps are utilized as practical tools to motivate women to return for their postpartum follow-up (82). Early studies on mobile application-based interventions showed promising results, but long-term effectiveness of mobile applications in postpartum GDM management is unclear (83). Much work is still needed to determine the effectiveness of mobile applications in engaging a broad audience with various levels of literacy and digital experience (83).

#### 2.2.4 Is postpartum OGTT enough to evaluate other metabolic risks?

Dyslipidemia is a physiological response in pregnancy driven by secretion of steroid hormone (e.g., progesterone), increased hepatic synthesis of triglycerides, and reduced lipoprotein lipase activity in adipose tissue (84). The characteristic finding at the 12th week of gestation is an elevated maternal triglyceride (TG) level and a mild increase in low-density lipoproteins (LDLs) and high-density lipoproteins (HDLs) (84). Altered lipid levels at 3 months postpartum (85) rarely normalise within a year after delivery (86–88). Of note, one in six women with abnormal glucose tolerance had an abnormal lipid profile postpartum, and one in four women with NGT had dyslipidemia (89). Another study reported 43% of women with GDM who had normoglycaemia at 6 months postpartum had dyslipidaemia (90). Dyslipidemia during and after pregnancy (88) aggravated endothelial dysfunction and promoted premature atherosclerosis (91), leading to increased CVD events per 10,000 person-years in women with GDM compared to those without (5.8 vs. 2.5, *p* < 0.0001) (88). CVD events could occur in a subset of women with GDM who did not develop intercurrent T2DM (3.2 vs. 2.2, *p* < 0.0001) (92). In these women, mediation analysis showed that HDL, triglycerides and LDL cholesterol (without glycaemia) contributed to elevated CVD risk at 40.8% 12.1% and 9.9%, respectively (92).

CVD monitoring and modification of CVD risk are thus critically needed in women with GDM after pregnancy. However, surveillance protocols for CVD have been mostly focused on individuals aged 40–80 years with T2DM and not on younger women with GDM (93). Females of reproductive age are less likely to be offered statin, and even if offered, they are less likely to comply (94). Therefore, future research should consider intervention strategies to reduce progression of atherosclerotic disease in women with GDM, beyond preserving the β-cell function.

### 2.3 Challenges in implementing postpartum interventions

#### 2.3.1 Decision on the most appropriate postpartum intervention

Currently, the most appropriate lifestyle intervention to prevent diabetes during postpartum period is not known. The Diabetes Prevention Program (DPP) and the Finnish Diabetes Prevention Study (FDPS) have shown that lifestyle interventions were effective in reducing risk of T2DM by ∼58% in women with a history of GDM (95, 96) and in at risk non-pregnant individuals (97). However, other lifestyle intervention trials during pregnancy did not show changes in fasting glucose or insulin sensitivity (98, 99). Women enrolled in the Tianjin Gestational Diabetes Mellitus Prevention Program, had significant weight loss and reduction in plasma insulin levels in the lifestyle intervention arm compared to the control group during the first year (100) but it is unclear if these effects were sustained (101). A systematic review on lifestyle intervention conducted in at-risk population in lower-middle income countries (LMIC) showed a possible reduction in T2DM incidence by 25% but the type of lifestyle intervention was heterogenous (102).

Various factors could impact on the success of a diabetes prevention program. Besides the type of intervention (physical activity or dietary changes or both), the level of intensity of contact between the healthcare worker and women, the mode of contact and whether the trial design included patients with prior education or elements of behavioural therapy such as goal setting, stimulus control and motivational interview could influence outcomes. Participants in the DPP received 16-sessions (6 months) of intensive curriculum on behavioral change (103) to reach a 58% reduction in diabetes risk (95). In the Mothers After Gestational Diabetes in Australia Diabetes Program, a 12-months intervention consisting of program handbook, face-to-face and telephone follow up calls ensured participants achieve their health goals (104). Latino women with GDM received an 8-weeks culturally appropriate education classes and monthly support sessions over a 6-months period to sustain health behaviour change (105). In South Asian population (India, Sri Lanka and Bangladesh), a 12-months lifestyle intervention trial on diet and physical activity did not yield any change in glycaemic status at 14 months in women with GDM (106). The South Asian ethnic group is likely to have a different trajectory for developing dysglycaemia during the postpartum period. Thus, a cultural and country specific approach is clearly needed to implement diabetes prevention care after delivery (106).

Cost-effectiveness is an important factor to consider in the implementation of prevention programs for women with GDM. Unfortunately, few studies studied the cost-effectiveness of T2DM prevention in women with GDM. Werbrouck et al. concluded that an OGTT every three years could potentially lead to the lowest cost per T2DM case detected (107) but the modelling studies done were 14–30 years ago (1993–2010) and did not include incremental analysis or a comparator population of “no screening/prevention” (107). No further randomized controlled trials on the cost-effectiveness of lifestyle intervention programs has since been conducted (108), representing a clear research gap in women’s health.

Metformin and Troglitazone were studied as potential agents to reduce the risk of diabetes in women with previous GDM. Compared to the placebo, women with previous GDM (*n* = 350) benefited from metformin and intensive lifestyle modification, with both these interventions achieving a ∼50% and ∼53% risk reduction of diabetes, respectively (95). The effect of metformin or lifestyle intervention also persisted for 15 years in DPP study (109). Likewise, in the Troglitazone in Prevention of Diabetes (TIRPOD) study, treatment with Troglitazone (400 mg per day) in 133 women with GDM of Hispanic origin for 30 months resulted in more than 50% reduction in the incidence rate of T2DM (12.1% in Troglitazone vs. 5.4% in placebo group, *P* = 0.03) (110). Two-thirds of the women receiving Troglitazone had improved insulin sensitivity and a greater mean decrease in fasting glucose (110) and protection against diabetes for 8 months after stopping therapy (110). Due to concerns about hepatotoxicity, troglitazone was discontinued. Dipeptidyl-peptidase IV (DPPIV) inhibitors and sodium-glucose co-transporter 2 (SGLT2) inhibitors were studied in small number of patients with previous GDM. A proof-of-concept study in forty women with prior GDM showed that a 16-weeks treatment with metformin and sitagliptin significantly increased first-phase insulin secretion from 720.3 ± 299.0 to 995.5 ± 370.3 pmol/L (*P* = 0.02) but no significant change was observed with sitagliptin or metformin alone (111). In another study, women with previous GDM lost 4.9% of their original weight after 24 months of dapagliflozin-metformin combination compared to metformin (1.4% weight loss) or dapagliflozin alone (3.2%) (111). Women with prior GDM randomized to 84-weeks of metformin 2000 mg and liraglutide 1.8 mg subcutaneously per day had improved postpartum insulin sensitivity and reduced body weight compared to women receiving metformin alone (112). More studies are clearly needed to establish the optimal early postpartum treatment for this high-risk young cohort.

#### 2.3.2 Implementation of care in high-income (HIC) and low middle-income (LMIC) countries

Challenges faced in implementing postpartum GDM care are contextual and highly dependent on the societal/cultural barriers and health system resources available for maternal care in each country. Postpartum care for women with GDM in high income countries (HIC) is at present, suboptimal (66). On an individual level, the barriers identified in HIC include fear of diagnosis of diabetes, inadequate information on postpartum care, difficulties in adhering to a healthy lifestyle long term (60, 113–117). From a health system perspective (60), challenges perceived are lack of concern on postpartum health by policy makers (67), lack of agreed quality and accountability measures between providers and patients on a global/local level (66, 118). Most countries by default, would refer women with GDM to primary care as a standard practice but quality of postpartum care in each practice varies (20, 21, 119). In Finland, a universal healthcare system exists to provide a series of intervention from primary care to preventive care and through to treatment for women with GDM (120). However, even in Finland, return rate for postpartum OGTT testing ranges from 30.9%–85.2%, with higher rates of return in areas that offer lifestyle intervention (121). In the United States, continuous care to pregnant women with or without GDM during the postpartum period depends on whether the women were enrolled in health systems that offer prevention programs (122). In Australia, postpartum care depends on whether the woman is followed up in a public or private sector (123). Those receiving postpartum OGTT test in a public sector are likely to have fragmented care due to inadequate staffing, difficulty in establishing a continuity of care after delivery (123) while those in private sectors are more likely to be enrolled in a long-term follow up programme (123).

The data on postpartum care for women with GDM in LMIC are limited, compared to HIC (66, 118, 124). Some of the challenges identified in LMIC are similar to those seen in HIC (e.g., fear/anxiety about the perceived diagnosis of overt diabetes) (125, 126). However, the more pertinent issues are associated with social and cultural issues and differences in health systems between countries (60, 118, 124, 127). Shortage of trained healthcare professionals (118), issues with transportation to health centres (128) or lack of financial means to see a healthcare professional and poor understanding on implication of GDM on long term metabolic health (125, 129) are highlighted as barriers to postpartum follow up (127). The lack of robust follow-up systems (124), guidelines or glucose equipment for postpartum care (118) pose substantial barriers to screening and counselling. Healthcare professionals in LMIC such as India or Turkey often do not recommend women with GDM to have postpartum testing according to latest evidence (130, 131). It is therefore not surprising that fewer than one in ten people with diabetes in LMIC receive the standard level of care as detailed in international guidelines (132). In LMIC, inadequate collaboration between different specialists impairs the process of coordinating care for women (124). Women often have to consult different services and specialists and the delays experienced in receiving care increases the risk of drop-outs (124).

Society and cultural factors influence the provision of care. In Southeast China (133) or Vietnam (134), GDM is perceived by women or family members as an insignificant condition that disappears after delivery and this greatly influence their care-seeking behaviour (134). Husbands’ approvals are sometimes needed before a woman seek for medical care (124). Illiteracy and the cultural expectation for woman to deliver at home results in missed opportunities to educate women and family (118). In Tonga, physical activity as a preventive measure is perceived as a “foreign” concept, resulting in a reluctance to engage in physical activity measures after delivery (135). Although society and cultural issues emerge as a prevailing factor in shaping care in LMIC, factors such as low perceived importance of postpartum GDM care by policy makers (66, 67, 118, 127), absence of financing strategies and disorganized care processes remain a common issue globally (129, 133, 134). Despite these issues, delivery of postpartum care is still possible if innovative, country-specific and culturally appropriate methods are carried out (see below) (58, 79, 136, 137).

Medical specialisation has continued to expand in LMIC but the type and number of specialists available to deliver care in a particular field may not necessarily translate to improved service availability (138). Factors like inadequate incentivisation and career advancement opportunities for specialists in public sector often lead to migration of specialists from public to private sector, which influence delivery of equitable public health services (138). Thus, country-specific policies should be in place to determine the level of health systems that require specialists’ involvement (138). Public health services data in Iran and China showed that community health workers could play a beneficial role in coaching, hypertension and diabetes prevention (139, 140). In Nepal and India, early preliminary studies suggest that mobile or tablet-based electronic decision support systems led by health workers could support patient education and improve screening and management of GDM (141, 142). A good example of success is the Women in India with GDM Strategy Project (WINGS) in Southern India (136) which showed improved GDM complications rate (79), postpartum follow up and a reversal of trend of declining physical activity associated with pregnancy with low-cost intervention *n* (137). Innovative measures used include having trained health workers educate on nutrition through cooking demonstrations (130, 136) or via a diet and nutrition “snakes and ladders” game (136), providing women with GDM a nutrition booklet (136) and a pedometer to increase daily step count (137), and contacting women to remind them to return for postpartum follow up (79).

## 3 Conclusion

Management of women with GDM has conventionally been focused on lowering the glycaemic excursion during pregnancy with the overarching aim of reducing pregnancy complications and fetal macrosomia. However, evidence suggests life-long metabolic sequalae of GDM impacts on a woman’s overall health (6, 8), and with this, the larger social construct. Despite this, care for women with GDM in the postpartum period is suboptimal. A seamless transition from obstetric care to primary care with an emphasis metabolic and cardiovascular health in women with GDM is currently non-existent. Thus, it is critical to recognize GDM as a double-edge sword, which presents as a risk to mother and child during antenatal period but also an opportunity to modify the progression to overt T2DM and CVD (143). This needs to occur in tandem with efforts from clinicians, policy makers and professional bodies. Whilst novel and emerging anti-diabetic medications could offer promise, this risk is unlikely to be fully mitigated if efforts are not made to engage, educate and empower these “high-risk” women. A system level change is required to facilitate transfer of medical information between healthcare professionals and community, and this should occur in parallel with social support programs that promote lifestyle intervention to promote a global shift in healthcare beliefs and practice. Women with previous GDM are in the most productive years of their lives, not limiting to economy contribution and family building. Evidently, an orchestrated program of care amongst different specialists and various domains is urgently needed to improve women’s health. There is clearly much work to be done before we could bridge evidence into clinical practice but overcoming the obstacles ahead is a necessary step to realise a future of diminished diabetes risk in women with GDM and their future generations.
